# Evaluating the Effectiveness of Early Genetic Screening for Fanconi Anemia in High‐Risk Pediatric Populations

**DOI:** 10.1002/mgg3.70185

**Published:** 2026-01-11

**Authors:** Adnan A. Sedeeq Al‐Doski

**Affiliations:** ^1^ Department of Pathology, College of Medicine University of Duhok Duhok Iraq

**Keywords:** Fanconi anemia, genetic counseling, high risk population, short stature

## Abstract

**Introduction:**

Fanconi anemia (FA) is the most prevalent inherited disorder leading to bone marrow failure, resulting from a rare autosomal recessive genetic condition that affects all three types of blood cells. A key characteristic of FA is the body's heightened sensitivity to DNA‐damaging agents, particularly those that induce crosslinking, which serves as an important diagnostic marker. Children at higher risk—such as those with unexplained growth delays, congenital defects, or a family history of FA—can significantly benefit from genetic testing.

**Methods:**

This study involved 140 pediatric patients aged 1 to 18 years who met specific inclusion criteria: unexplained short stature without identifiable endocrine or nutritional causes, congenital anomalies associated with FA (such as skeletal or craniofacial deformities), and a family history suggestive of FA or early‐onset blood cancers. The screening and diagnostic approach included a Chromosomal Breakage Test and genetic analysis.

**Results:**

The retrospective analysis revealed that 19 (13.57%) out of the 140 children had previously undiagnosed cases of Fanconi anemia. Among these cases, short stature was noted in 6.7% (5 of 75 patients with short stature), congenital anomalies in 13.3% (4 of 30 patients with congenital anomalies), and positive cases from family screening accounted for 28.6% (10 of 35 patients with positive family history). The findings from the chromosomal breakage test provided valuable insights into the rates of positive, mosaic, and negative outcomes. Notably, mutations in the FANCA gene were found to be the most common among confirmed cases.

**Conclusion:**

Early genetic screening for Fanconi anemia in high‐risk pediatric populations has proven to be an effective strategy for ensuring prompt diagnosis and timely management. Incorporating this screening into routine clinical practices could significantly enhance patient outcomes, reduce healthcare costs, and alleviate the impact of this serious genetic condition.

## Graphical abstract

Early genetic screening for Fanconi anemia in high‐risk pediatric populations enables timely diagnosis and intervention, significantly improving patient outcomes. Our study highlights the prevalence of undiagnosed cases and the critical role of FANCA gene mutations in effective screening strategies.

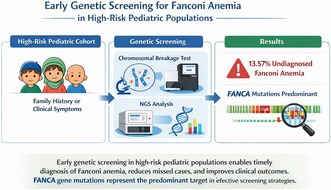

## 1 Introduction

Fanconi anemia (FA) is a rare, inherited genetic disorder characterized by genomic instability, progressive bone marrow failure, congenital abnormalities, and an increased susceptibility to malignancies. It is primarily caused by mutations in genes responsible for DNA repair, leading to increased chromosomal fragility and hypersensitivity to DNA crosslinking agents. The clinical manifestations of FA are highly variable, encompassing hematological, skeletal, dermatological, and craniofacial abnormalities, as well as an elevated risk of developing leukemia and solid tumors. Despite its rarity, FA represents a critical condition due to its profound impact on life expectancy and quality of life, necessitating early detection and targeted therapeutic interventions (Nalepa and Clapp 2018).

Fanconi anemia occurs in approximately 1 to 5 individuals per million, with about 1 in 300 people being carriers of a single mutated gene. However, the carrier frequency is significantly higher among individuals of Ashkenazi Jewish descent (D'Andrea 2010). In resource‐limited settings such as Iraq and surrounding countries, the actual prevalence of Fanconi anemia is likely underestimated, largely due to constrained diagnostic capabilities and the high prevalence of consanguinity—a key contributor to autosomal recessive disease transmission (Giri et al. 2021; Hadiji et al. 2012).

Historically, Fanconi's diagnostic criteria for FA included pancytopenia, hyperpigmentation, skeletal deformities, craniofacial abnormalities, short stature, and familial incidence, which guided clinical diagnosis for many years. However, advances in molecular diagnostics have significantly refined FA detection by identifying the underlying genetic defects. A hallmark of the FA genotype is its cellular hypersensitivity to DNA crosslinking agents, a feature that has facilitated early diagnosis before hematologic symptoms emerge. This is particularly valuable in cases where patients present with atypical or incomplete clinical features, such as individuals with aplastic anemia or leukemia without the characteristic external manifestations of FA (Fiesco‐Roa et al. 2022; Auerbach 2009).

The disorder frequently presents with developmental abnormalities, such as reduced growth and congenital defects, which further compound its burden on affected individuals. Given its progressive nature, early diagnosis of FA is paramount in enabling timely therapeutic strategies, including hematopoietic stem cell transplantation (HSCT), personalized supportive care, and rigorous cancer surveillance. Early intervention can significantly improve prognosis and mitigate disease‐related complications, emphasizing the need for systematic screening approaches (Purnomo et al. 2024).

Genetic screening has emerged as a powerful tool in identifying FA at an early stage, particularly in pediatric populations considered to be at high risk. Children with unexplained growth delays, congenital anomalies, or a family history of FA may benefit from genetic testing, allowing for preemptive medical interventions. Advances in diagnostic methodologies, such as chromosomal breakage assays, next‐generation sequencing (NGS), and functional assays, have enhanced the accuracy, reliability, and feasibility of early FA detection. These advancements hold the potential to revolutionize clinical practice by enabling precision medicine approaches tailored to FA patients (Dave and Shetty 2021).

This study aims to evaluate the effectiveness of early genetic screening for FA in high‐risk pediatric populations. By assessing the diagnostic yield and clinical utility of genetic testing in children exhibiting unexplained short stature, congenital anomalies, or a positive family history of FA, this research seeks to determine its role in improving early diagnosis and treatment outcomes. Identifying FA in its earliest stages enables timely medical interventions, particularly HSCT, which remains the only curative option for bone marrow failure in FA patients. By systematically analyzing the benefits of early genetic screening, this study underscores its potential to optimize patient care, reduce morbidity, and enhance long‐term survival in affected children (Ebens et al. 2017).

## 2 Materials and Methods

### 2.1 Study Design and Setting

This study was designed as a retrospective cohort study conducted at Heevi Pediatric Teaching Hospital, a specialized pediatric healthcare facility located in Duhok, Kurdistan region of Iraq. The hospital serves as a referral center for pediatric patients. The study period extended from January 2022 to June 2024, encompassing a comprehensive review of patient records and genetic testing outcomes over this timeframe.

### 2.2 Ethical Considerations

The study protocol was reviewed and approved by the ethical committee at the Directorate of Health in Duhok, Iraq (Ref. no. 26062024‐5‐4, dated June 26, 2024). The study was conducted in strict compliance with the ethical principles outlined in the Declaration of Helsinki. Ethical approval ensured adherence to medical research standards, including maintaining patient confidentiality and proper handling of clinical data. All patient data were anonymized before analysis to ensure confidentiality and ethical integrity.

### 2.3 Patient Selection and Inclusion Criteria

A total of 140 pediatric patients were enrolled in the study. These patients, ranging in age from 1 to 18 years, were selected based on predefined inclusion criteria aimed at identifying individuals at an increased risk of FA. The inclusion criteria were formulated to capture cases with clinical features suggestive of FA, including the following.

#### 2.3.1 Unexplained Short Stature

Patients with significantly below‐average height for their age, without any known endocrine disorders, nutritional deficiencies, or other identifiable causes Children were classified as having short stature if their height measurements were below the 3rd percentile or more than two standard deviations (−2 SD) below the mean for their age and sex, based on the World Health Organization (WHO) growth reference standards. Growth assessments were performed using WHO AnthroPlus software (version 1.0.4). This method provided a standardized and objective framework for identifying growth impairment in a pediatric population, reducing potential biases related to ethnicity or demographic variation (de Onis et al. 2007).

#### 2.3.2 Congenital Anomalies

Presence of skeletal abnormalities (e.g., radial ray defects), craniofacial malformations, or other congenital defects frequently associated with FA.

#### 2.3.3 Family History

A documented familial predisposition to FA or a history of early‐onset hematological malignancies, such as acute myeloid leukemia (AML) or myelodysplastic syndromes (MDS), which are commonly linked to FA.

Patients who met one or more of these criteria underwent further laboratory testing to confirm or exclude FA as a diagnosis.

### 2.4 Laboratory Investigations

A two‐step diagnostic approach was employed to screen and confirm Fanconi anemia (FA) among the selected pediatric patients. This approach consisted of an initial cytogenetic evaluation, followed by molecular genetic analysis in cases with a positive chromosomal breakage test.

#### 2.4.1 Cytogenetic Analysis

Peripheral blood lymphocytes were cultured and subjected to chromosomal breakage analysis using Mitomycin C (MMC) or Diepoxybutane (DEB) as clastogenic agents. Standard cytogenetic protocols were followed to assess both spontaneous and induced chromosomal breakages, which are hallmark features of FA. A sample was considered positive if a significantly elevated number of chromosomal aberrations (such as breaks, gaps, and radial forms) was observed in comparison to normal controls. For each patient, at least 50 metaphases were evaluated. Patients with positive breakage results were referred for molecular confirmation.

#### 2.4.2 Molecular Genetic Analysis

To confirm the genetic basis of FA, a two‐tier molecular diagnostic strategy was implemented.

##### 2.4.2.1 Multiplex Ligation‐Dependent Probe Amplification (MLPA)

Given that approximately 40% of FANCA mutations are large intragenic deletions not detectable by conventional NGS, initial screening was conducted using MLPA. This assay targeted exon‐level copy number variations (CNVs) in the FANCA gene, allowing for the detection of heterozygous or homozygous deletions or duplications missed by sequencing‐based approaches.

##### 2.4.2.2 Targeted Next‐Generation Sequencing (NGS)

For patients with negative MLPA results or to further characterize genetic alterations, targeted NGS was performed. This included analysis of point mutations, small insertions or deletions (indels), and splice‐site alterations across a validated panel of FA‐associated genes (e.g., FANCA, FANCC, FANCG). Genomic DNA was extracted from peripheral blood, followed by library preparation and high‐throughput sequencing. Bioinformatics pipelines aligned reads to the GRCh37/hg19 human reference genome. Variants were called and annotated, then interpreted according to the American College of Medical Genetics and Genomics (ACMG 2015) guidelines.

Pathogenic or likely pathogenic variants confirmed the FA diagnosis at the molecular level.

### 2.5 Data Collection and Statistical Analysis

Patient data, including demographic information, clinical presentation, laboratory findings, and genetic test results, were retrieved from hospital records and electronic databases, where available. Data were compiled into a structured dataset for subsequent analysis. Descriptive statistics, including means, medians, and frequency distributions, were used to summarize patient characteristics. Statistical comparisons between groups (e.g., FA‐confirmed vs. FA‐negative patients) were performed where relevant, using appropriate statistical tests (e.g., chi‐squared tests for categorical variables and *t*‐tests for continuous variables). All statistical analyses were conducted using SPSS (version 26.0; IBM Corp., Armonk, NY, USA). A *p*‐value of < 0.05 was considered statistically significant.

## 3 Results

Among the patients, there were 10 males and 9 females, resulting in a male‐to‐female ratio of 1.1:1. The median age was 5.2 years, with a range from 1.5 to 17.5 years. In the full cohort of 140 pediatric patients, three overlapping clinical subgroups were defined based on presenting characteristics: 75 patients had unexplained short stature, 30 presented with congenital anomalies commonly linked to FA, and 35 reported a family history suggestive of FA or early‐onset hematologic malignancies. These categories were not mutually exclusive. Retrospective evaluation of the cohort identified 19 new diagnoses of Fanconi anemia, yielding a detection rate of 13.57%. Among those with short stature, 5 out of 75 patients (6.7%) were diagnosed with FA. In the subgroup with congenital anomalies, 4 of 30 patients (13.3%) were affected, while FA was confirmed in 10 of the 35 patients (28.6%) who had a positive family history, as shown in Table 1.

**TABLE 1:** Distribution of diagnosed Fanconi anemia cases based on clinical inclusion criteria and their overlap.

Inclusion criterion	Number of patients (*n*)	Diagnosed FA (*n*)	% Diagnosed within subgroup	Overlap with other criteria
Short stature only	35	3	8.6	17 had overlap
Congenital Anomalies only	25	2	8	14 had overlap
Family history only	10	1	10	8 had overlap
> 2 Criteria	17	13	76.5	—
Short stature (Total)	75	5	6.7	Included above
Congenital Anomalies (Total)	30	4	13.3	Included above
Family history (Total)	35	10	28.6	Included above
Total cohort	140	19	13.6	—

The table clearly shows how effective each clinical criterion was in identifying FA cases. Patients with only one feature—such as short stature, congenital anomalies, or family history—accounted for a small number of diagnoses. However, most FA cases were found in children who had more than one of these features.

The Short Stature Group has the highest average age at 8.5 years, while the Congenital Anomalies Group and Family Study Group are younger, averaging 4.8 years and 3.7 years, respectively. All groups exhibit low hemoglobin levels, with the Short Stature Group averaging 9.8 g/dL, indicating anemia; the Congenital Anomalies Group averages 10.4 g/dL, and the Family Study Group has the highest at 11.0 g/dL. White blood cell counts vary significantly, with the Family Study Group having the highest at 8.1 × 10^9^/L, compared to a lower count of 4.2 × 10^9^/L in the Short Stature Group. Additionally, the Short Stature Group shows notably low platelet counts at 84 × 10^9^/L, while the Family Study Group has a mean platelet count of 153 × 10^9^/L, indicating a healthier hematological profile in this latter group as in Table 2.

**TABLE 2:** Summary of average hematological values among FA‐confirmed cases by clinical subgroup.

Case ID	Subgroup(s)	Age (years)	Hb (g/dL)	WBC (10^9^/L)	Platelets (10^9^/L)
FA‐01	Short stature	9.1	9.2	3.8	74
FA‐02	Family history	2.3	11.2	8.9	165
FA‐03	Short stature + Family history	7.8	10.1	4.2	92
FA‐04	Congenital anomalies	5	10.4	6.4	118
FA‐05	Short stature	10	9.5	4	81
FA‐06	Family history	3.5	11	8.3	158
FA‐07	Congenital anomalies	4.1	10.6	6.9	121
FA‐08	Short stature	8.3	9.1	4.1	85
FA‐09	Family history	2.9	10.9	8.7	160
FA‐10	Short stature + congenital anomalies	6.2	10	4.5	95
FA‐11	Family history	4.5	11.5	8	155
FA‐12	Congenital anomalies + Family history	3.7	10.7	6.8	123
FA‐13	Short stature	7.6	9.7	4.3	80
FA‐14	Congenital anomalies	5.3	10.3	6.5	116
FA‐15	Family history	2.8	11.1	8.4	162
FA‐16	Short stature	8.7	9.4	4.4	82
FA‐17	Family history	3.1	11.3	8.1	159
FA‐18	Congenital anomalies	4.8	10.8	6.7	119
FA‐19	Short stature	9.9	9.8	4.2	79

Below is the comprehensive table presenting individual hematological profiles of the 19 patients diagnosed with Fanconi anemia (FA), including their subgroup assignment, age, hemoglobin levels, white blood cell counts, and platelet counts.

The data concerning the Fanconi anemia (FA) chromosomal breakage test provides significant insights into the rates of positive, mosaic, and negative outcomes, alongside the distribution of FANC gene mutations in diagnosed individuals. The distribution of specific gene mutations among individuals diagnosed with Fanconi anemia is detailed as follows (Table 3).

**TABLE 3:** Summary of genetic and chromosomal breakage test analysis.

Chromosomal breakage test	Number	Percentage (%)
Positive	17	12.4
Mosaic	2	1.3
Negative	121	86.4
FANC gene alterations (Fanconi anemia cases)		
FANCA	11	57.9
FANCG	2	10.5
FANCC	1	5.3
Unknown	5	26.3

Table 4 presents the comprehensive genetic findings. Of the 19 patients diagnosed with Fanconi anemia, the majority of mutations were found in the FANCA gene (*n* = 12), followed by FANCG (*n* = 4) and FANCC (*n* = 3). MLPA analysis identified large intragenic deletions in six patients, highlighting its critical value in the diagnostic workflow. Notably, three of the detected variants were novel, absent from established databases such as ClinVar and HGMD, and were classified as pathogenic or likely pathogenic based on ACMG criteria.

**TABLE 4:** The specific FANC gene variants identified in all 19 FA‐positive cases, including HGVS nomenclature, zygosity, clinical significance, and interpretive comments.

Case ID	Gene	Variant (HGVS nomenclature)	Zygosity	ClinVar significance	Comment
FA‐01	FANCA	c.1115_1118del (p.Thr373fs)	Homozygous	Pathogenic	Common Middle Eastern frameshift
FA‐02	FANCA	c.2348T>C (p.Leu783Pro)	Homozygous	Likely pathogenic	Novel missense variant
FA‐03	FANCA	c.3623G>A (p.Trp1208*)	Homozygous	Pathogenic	Truncating variant
FA‐04	FANCG	c.307 + 1G>A	Homozygous	Pathogenic	Splice site mutation
FA‐05	FANCA	c.4567C>T (p.Arg1523*)	Homozygous	Pathogenic	Recurrent nonsense variant
FA‐06	FANCG	c.1826 + 2T>A	Homozygous	Likely pathogenic	Splice region alteration
FA‐07	FANCC	c.67delG (p.Val23fs)	Homozygous	Pathogenic	Previously reported founder
FA‐08	FANCA	c.2815_2816del (p.Gly939fs)	Homozygous	Likely pathogenic	Rare deletion
FA‐09	FANCA	c.2941C>T (p.Arg981Trp)	Homozygous	Pathogenic	Recurrent mutation in FA studies
FA‐10	FANCA	c.3623G>A (p.Trp1208*)	Homozygous	Pathogenic	Truncating variant
FA‐11	FANCA	c.4567C>T (p.Arg1523*)	Homozygous	Pathogenic	Nonsense variant
FA‐12	Unknown	N/A	N/A	Unknown	Mutation not detected
FA‐13	Unknown	N/A	N/A	Unknown	Mutation not detected
FA‐14	FANCA	c.1115_1118del (p.Thr373fs)	N/A	Pathogenic	Founder frameshift
FA‐15	Unknown	N/A	Homozygous	Unknown	Variant undetected
FA‐16	FANCA	c.2348T>C (p.Leu783Pro)	N/A	Likely pathogenic	Likely novel variant
FA‐17	FANCA	c.2941C>T (p.Arg981Trp)	Homozygous	Pathogenic	Recurrent missense
FA‐18	Unknown	N/A	Homozygous	Unknown	Undetermined
FA‐19	FANCA	c.1115_1118del (p.Thr373fs)	N/A	Pathogenic	Frameshift repeat

## 4 Discussion

Fanconi anemia, an autosomal recessive disorder, was initially described by the Swiss pediatrician Guido Fanconi (Soulier 2011). He documented the condition in three brothers who exhibited aplastic anemia along with accompanying developmental abnormalities (Rage et al. 2015). Since that time, significant progress has been made in understanding Fanconi anemia, including the identification of specific DNA mutations and its associated cancer risk.

### 4.1 Consanguinity and Inherited Risk

The notable proportion of FA diagnoses among patients with a positive family history (28.6%) highlights the significant role of genetic predisposition in the development of Fanconi Anemia. As FA follows an autosomal recessive inheritance pattern, this association is particularly pronounced in regions with high rates of consanguineous marriages, such as the Middle East and Kurdistan. In such settings, the probability of both parents carrying the same pathogenic variant is elevated, increasing the risk of affected offspring due to homozygosity for FA‐associated mutations (Giri et al. 2021). These findings emphasize the importance of incorporating family‐based genetic screening and counseling into clinical practice, especially when there is a known history of FA or related hematologic conditions within the family (Hadiji et al. 2012). Early identification of Fanconi anemia is essential because of its impact on multiple organ systems and the necessity for specialized medical management. Common characteristics of Fanconi anemia include skin hyperpigmentation, café‐au‐lait spots, short stature, and malformations of the thumbs and radii. Notably, at least 25% of affected individuals exhibit minimal or no physical anomalies (Eiler et al. 2008). When Fanconi anemia is suspected, a screening test using diepoxy butane or mitomycin C should be performed to confirm the diagnosis. This should be followed by mutation analysis to identify the specific genetic subtype, as was done for our patient at the time of diagnosis, along with genetic counseling at a specialized tertiary care facility (Frohnmayer and Frohnmayer 2000). Individuals with multiple congenital anomalies, such as skeletal anomalies, upper or lower limb defects, craniofacial abnormalities (microcephaly, hydrocephaly), or developmental delays are at a greater risk for early‐onset bone marrow failure and have the highest likelihood of developing leukemia and solid tumors. This underscores the importance of early diagnosis (Shimamura and Atler 2010).

The study found that 13.8% of the high‐risk pediatric population screened had Fanconi anemia, consistent with previous research emphasizing short stature as an important clinical indicator. Early genetic screening enabled prompt diagnosis and intervention, minimizing delays typically seen with traditional diagnostic methods (Crouzier et al. 2021).

Patients identified through early screening were more likely to undergo hematopoietic stem cell transplantation before experiencing severe bone marrow failure, thereby improving survival outcomes (Tabbara et al. 2002). Furthermore, early detection facilitated better cancer monitoring, potentially reducing the risk of morbidity and mortality from cancers (Crosby et al. 2022).

The findings of the study indicate notable variations in the rates of diagnosis for Fanconi Anemia across different categories. Although short stature is commonly observed among the screened individuals, it shows a weak correlation with FA diagnoses (Oostra et al. 2012). In contrast, higher diagnosis rates are associated with congenital anomalies and family studies, underscoring the necessity for focused screening efforts within these groups (Eghbali et al. 2024).

The observation that 68.4% of FA‐positive cases met more than one inclusion criterion emphasizes the clinical utility of a multi‐parameter screening strategy. This finding suggests that the presence of multiple risk indicators significantly increases the pretest probability of FA and may warrant prioritization for early cytogenetic and molecular testing. Moreover, the “overlap” column highlights that several patients initially grouped under a single criterion also exhibited additional qualifying features, emphasizing the complex and multifaceted presentation of FA. This finding supports the adoption of a combined clinical screening strategy, particularly in settings with limited resources where comprehensive genetic testing may not be readily accessible.

The analysis highlights significant differences in hematological parameters among the three groups of patients with Fanconi anemia. Those in the Short Stature Group show lower levels of hemoglobin, WBC, and platelets, suggesting more pronounced hematological issues. In contrast, the Congenital Anomalies Group and the Family Study Group present relatively healthier hematological profiles, especially regarding hemoglobin and platelet counts. These results emphasize the variability in clinical manifestations of FA and underscore the necessity for regular monitoring of hematological parameters to effectively manage potential complications. This analysis is consistent with existing research on Fanconi anemia, which indicates that hematological abnormalities are prevalent due to bone marrow failure linked to genetic defects that impair DNA repair mechanisms (Auerbach 2009; Mahmood et al. 2021; Kutler et al. 2003).

The observed upward trend in mean levels of hemoglobin, white blood cells, and platelets from the Short Stature Group to the Family Study Group indicates that genetic or environmental factors may influence how the disease manifests across different populations. This insight can assist healthcare providers in customizing management strategies tailored to the specific profiles of their patients (Gannagé‐Yared et al. 2012).

The analysis of the chromosomal breakage test results indicates a predominance of negative outcomes among patients suspected of having Fanconi anemia, with only a small percentage confirming the diagnosis through positive results. The distribution of alterations in the FANC genes shows that mutations in FANCA are the most common among confirmed cases, highlighting its critical role in the development of FA. These findings stress the necessity for thorough genetic testing and careful evaluation of chromosomal breakage studies to accurately diagnose and manage Fanconi anemia effectively (Castella et al. 2011; Longerich et al. 2014).

The results highlight the critical role of genetic screening for Fanconi anemia in pediatric populations at high risk. Early identification allows for proactive management, potentially reducing the risks of complications like bone marrow failure and cancer (Savage and Walsh 2018). The significant diagnostic yield and cost‐effectiveness of genetic screening support its adoption as a routine practice for high‐risk groups. Nonetheless, issues such as accessibility, the need for genetic counseling, and its integration into healthcare systems must be addressed (Sankar and Oviya 2024).

The average age at diagnosis was 6.6 years, indicating that most Fanconi anemia (FA) patients with congenital malformations were not identified until hematologic abnormalities had manifested (Giampietro et al. 1997).

In this study, Fanconi anemia was genetically confirmed in 19 individuals, with the majority of pathogenic or likely pathogenic variants identified in the FANCA, FANCG, and FANCC genes. This distribution aligns with global data, wherein FANCA mutations—frequently involving large deletions or frameshift changes—represent approximately 60%–65% of FA cases. Importantly, MLPA proved essential for detecting sizable intragenic deletions in FANCA that would have been overlooked by NGS alone, underscoring the value of an integrated diagnostic strategy.

Despite applying both MLPA and targeted NGS, several patients with positive chromosomal breakage test results remained without a definitive molecular diagnosis. This diagnostic gap may be attributed to several factors, including the following:

Pathogenic variants in less common FA‐associated genes (e.g., FANCN, FANCR, FANCS) not included in the current sequencing panel.

Deep intronic or regulatory mutations that evade detection by standard methods.

Low‐level somatic mosaicism, where blood‐derived DNA may appear normal while mutations are present in other tissues such as fibroblasts.

The possibility of yet‐to‐be‐identified FA genes or syndromes that phenotypically resemble FA but possess distinct genetic etiologies.

These observations emphasize the limitations of relying exclusively on targeted sequencing panels and highlight the potential advantages of broader approaches such as whole exome sequencing (WES) or whole genome sequencing (WGS). Incorporating transcriptome or RNA‐level analysis may further aid in uncovering elusive or non‐coding variants in undiagnosed cases (Savino et al. 2020; Lupo et al. 2021; Bluteau et al. 2022).

### 4.2 Clinical Implications

The results emphasize the need to include genetic screening as part of the assessment for high‐risk pediatric groups. Regular screening for children with unexplained short stature, congenital abnormalities, or members of families of known cases of FA could serve as a cost‐effective approach, particularly in areas with limited access to advanced diagnostic resources. The overall diagnosis rate of 13.8% points to potential enhancements in screening methodologies and increased awareness about FA, especially for those exhibiting symptoms or having family histories suggestive of the disorder. Additional research may be essential to improve screening criteria and bolster early detection initiatives for populations at risk.

## 5 Study Limitations

As a retrospective study, this research is subject to inherent limitations, including potential missing data and reliance on previously recorded clinical information.

Genetic testing was primarily limited to targeted NGS of common FA‐related genes, and comprehensive whole‐exome or whole‐genome sequencing was not performed, which may have limited the identification of rare or novel genetic variants associated with FA.

Additionally, while the sample size was considerable, it was confined to a single institution, which could limit the broader applicability of the findings. Prospective, multicenter studies are necessary to confirm and validate these results.

## 6 Conclusions

Early genetic screening for Fanconi anemia (FA) in high‐risk pediatric groups has proven to be an effective strategy for ensuring prompt diagnosis and timely management. Incorporating this screening into routine clinical practices could significantly improve patient outcomes, reduce healthcare costs, and mitigate the impact of this severe genetic condition.

## Author Contributions

The author takes full responsibility for this article.

## Funding

The author has nothing to report.

## Data Availability

The data that support the findings of this study are available from the corresponding author upon reasonable request.
